# The Development of an Automated Fluid Infusion Management System to Prevent Hypotension During General Anesthesia: A Randomized Clinical Trial

**DOI:** 10.3390/jcm14248952

**Published:** 2025-12-18

**Authors:** Yuka Matsuki, Yukie Mizuta, Shuko Matsuda, Koyo Nishio, Midoriko Higashi, Ken Yamaura, Kenji Shigemi

**Affiliations:** 1 Department of Anesthesiology and Reanimatology, University of Fukui Hospital, Fukui 910000, Japan; matshu@u-fukui.ac.jp (S.M.); koyo@u-fukui.ac.jp (K.N.); kshigemi@u-fukui.ac.jp (K.S.); 2 Department of Anesthesiology and Critical Care Medicine, Graduate School of Medical Sciences, Kyushu University, Fukuoka 819000, Japan; mizuta.yukie.330@m.kyushu-u.ac.jp (Y.M.); higashi.midoriko.976@m.kyushu-u.ac.jp (M.H.); yamaura.ken.361@m.kyushu-u.ac.jp (K.Y.); 3 Maizuru Municipal Hospital, Kyoto 624000, Japan

**Keywords:** automated fluid infusion, hypotension, stroke volume, effective arterial elastance

## Abstract

**Background/Objectives:** This study aimed to develop and evaluate an automated fluid infusion management system for preventing hypotension during general anesthesia. **Methods:** This study was a single-blind, randomized, non-inferiority, clinical trial. Seventy-nine patients undergoing surgery under general anesthesia were randomly assigned to either an automatic group or a manual group. In the automatic group, the infusion rate was automatically adjusted based on stroke volume (SV) and effective arterial elastance (Ea), whereas in the manual group, the attending anesthesiologist manually adjusted the infusion rate according to the Enhanced Recovery After Surgery (ERAS) protocol. The primary endpoint was the proportion of time during anesthesia that mean arterial pressure (Pm) was maintained at ≥65 mmHg. Secondary endpoints included the proportion of time the estimated stroke volume index (esSVI) was below the threshold, total fluid volume administered, total phenylephrine dose, urine output, blood loss, and average estimated stroke volume variation (esSVV). **Results:** The results demonstrated non-inferiority of the automatic group to the manual group in maintaining Pm ≥ 65 mmHg (automatic group: 82.0 ± 12.7%, manual group: 79.9 ± 15.7%; difference [automatic group−manual group]: 2.0 percentage points; one-sided 97.5% CI lower limit: −4.7%; non-inferiority margin: −5%). There were no significant differences between the groups in total fluid volume, phenylephrine dose, urine output, or blood loss. No severe adverse events or device-related adverse events were observed. **Conclusions:** The automated system maintained intraoperative blood pressure safely and effectively.

## 1. Introduction

Intraoperative hypotension is known to cause cerebral infarction and myocardial infarction during surgery and adversely affect postoperative outcomes. Specifically, it has been demonstrated that mean arterial pressure (MAP) falling below 55–65 mmHg during surgery is associated with an increased risk of complications [1,2]. However, during induction of general anesthesia, anesthesiologists are extremely busy with face mask ventilation, mouth opening, tracheal intubation, and other tasks, and they are often unable to respond appropriately and immediately to hypotension that occurs during this time. We have developed and are using in clinical practice an automatic administration device for propofol, remifentanil, and rocuronium. During induction of general anesthesia, anesthetic drugs are automatically administered according to a predetermined procedure [3,4], but artificial respiration must be performed by the anesthesiologist using both hands. We developed a device that automatically administers a predetermined vasopressor and automatically increases the infusion rate when hypotension occurs.

A closed-loop system for vasopressor and infusion administration was developed by Joosten et al. [5,6]. Targeting cases with moderate to high risk, they set a mean arterial pressure (MAP) target and used a closed-loop system with PID (Proportional–Integral–Derivative) control. For infusion, they combined this with a system programmed with ERAS (Enhanced Recovery After Surgery) algorithms to automatically administer bolus infusions. However, this has not yet reached a clinically practical stage.

We developed a device for low-risk ASA-PS-1-2 cases that immediately administers phenylephrine and accelerates fluid infusion when mean arterial pressure falls below 65 mmHg. This device was expected to shorten the duration of intraoperative hypotension. Therefore, using a robotic anesthesia system (AsisTIVA; Nihon Kohden) for general anesthesia, anesthesiologists compared the duration of hypotension with the duration of blood pressure below 65 mmHg when hypotension was shortened by this device. This demonstrated the non-inferiority of the device compared with the anesthesiologist.

## 2. Materials and Methods

### 2.1. Ethics Approval

All protocols were approved by the ethics committees of the University of Fukui Hospital and Kyushu University and were conducted in accordance with the Declaration of Helsinki. The trial was registered with the Japan Registry of Clinical Trials (jRCTs052220147). Written, informed consent for participation was obtained from the patients.

This was a collaborative, two-center, randomized, single-blind, parallel-group, comparative trial. The participants were 79 patients scheduled to undergo surgery under general anesthesia who met the following criteria: (1) males or females aged 20 years or older at the time of consent; (2) patients classified as ASA Physical Status (PS) 1–2 by the American Society of Anesthesiologists risk criteria scheduled to undergo surgery under general anesthesia; and (3) patients who received a thorough explanation of this study, fully understood it, and voluntarily provided written, informed consent However, patients meeting any of the following criteria were excluded: (1) patients with a history of hypersensitivity to propofol, remifentanil, rocuronium, or sugammadex; (2) cases in which non-invasive blood pressure measurement could not be performed during surgery; (3) cases involving cardiovascular surgery; (4) cases with supraventricular rhythm disturbances such as atrial fibrillation, atrial flutter, or sinus arrhythmia; (5) cases with an estimated blood loss of 1000 mL or more; (6) cases involving pregnancy, lactation, possible pregnancy, or planned pregnancy; and (7) cases deemed inappropriate for enrollment in the study by the principal investigator or sub-investigator.

### 2.2. Anesthesia Procedures

**Figure 1:**
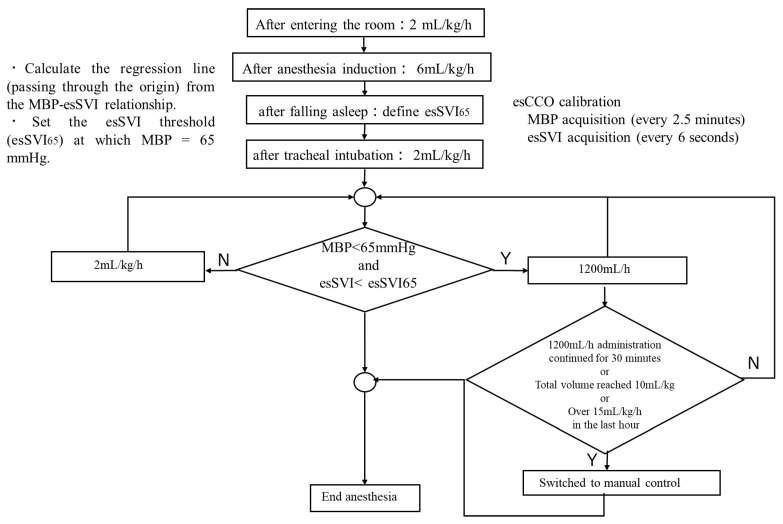
A Diagram summarizing the logic of the infusion algorithm. MBP: mean blood pressure; esCCO: estimated continuous cardiac output; esSVI: estimated stroke volume index.

Seventy-nine patients (ASA-PS 1,2) were randomly assigned to either the automatic group (A group) or the manual group (M group). No pre-anesthetic medication was used. After entering the operating room, patients received oxygen at 6 L/min via an oxygen mask. A non-invasive blood pressure (NIBP) cuff was attached to the upper arm, and electrodes for neuromuscular monitoring were placed on the forearm and hand. A bispectral index (BIS) monitoring system sensor (Medtronic, Minneapolis, MN, USA) was applied to the forehead, and BIS monitoring was initiated. In addition, a dedicated esCCO (estimated continuous cardiac output: Nihon Kohden, Tokyo, Japan) system sensor was attached to a fingertip for calibration, and hemodynamic parameters were measured along with transcutaneous oxygen saturation. Blood pressure was measured every 2.5 min. After oxygenation and denitrogenation, continuous administration of remifentanil was started using a robotic anesthesia system (AsisTIVA; Nihon Kohden, Tokyo, Japan). Subsequently, propofol infusion was started according to the sequential control of the robotic anesthesia system. Once the patient was asleep, the neuromuscular monitor was calibrated, followed by administration of rocuronium for muscle relaxation and tracheal intubation. Thereafter, the administration of propofol, remifentanil, and rocuronium was adjusted by the robotic anesthesia system, which performed closed-loop control to manage sedation, analgesia, and muscle relaxation with identical target values and measurement intervals, using the BIS value and train-of-four count (TOFC) as indicators in both groups. Therefore, this eliminates arbitrariness due to differences in anesthesia methods. The details of the closed-loop anesthesia delivery system have been described in detail in previous publications [3,4]. For fluid management, both groups received 1% glucose-added Ringer’s solution (Physio 140: Otsuka Pharmaceutical Factory, Tokushima, Japan). A diagram summarizing the logic of the infusion algorithm is shown in Figure 1. After entering the room, infusion commenced at 2 mL/kg/h and, after anesthesia induction, was increased to 6 mL/kg/h; then, after tracheal intubation up until the start of surgery, it was maintained at 2 mL/kg/h. In the M group, the attending anesthesiologist adjusted the infusion rate as deemed appropriate. In the A group, the infusion rate was adjusted based on SV. The threshold value of SV for adjusting the infusion rate was determined as follows: After falling asleep, the non-invasive continuously estimated stroke volume index (esSVI), normalized by mean arterial pressure (Pm) and body weight (BW), was measured three times to establish a linear relationship between Pm and esSVI. The slope was set as the initial value of effective arterial elastance (Ea) [7,8] (Equation (1)). Using this relationship, when Pm was 65 mmHg, the corresponding esSVI was defined as esSVI65, and this value was used as the lower threshold for SVI (Equation (2)).

Ea = Pm/esSVI(1)

esSVI65 = 65/Ea(2)

If esSVI was ≥esSVI65, the glucose-added Ringer’s solution was continuously infused at 2 mL/kg/h. If both the mean arterial pressure dropped below 65 mmHg and esSVI fell below esSVI65, infusion was started at 1200 mL/h. In both groups, when mean arterial pressure fell below 65 mmHg, phenylephrine 100 µg was automatically administered. If this was administered five consecutive times, subsequent blood pressure management was switched to manual administration. In addition, if infusion at 1200 mL/h continued for more than 30 min, or the total infusion volume reached 10 mL/kg, or the total hourly infusion volume reached 15 mL/kg, automatic infusion was discontinued and switched to manual control. As a continuous assessment, automatic vasopressor control was discontinued after five consecutive low Pm readings and switched to manual control. In this way, fluid overload with respect to autonomic nervous system fluctuations during a short time was avoided, and fluid replacement was selected only for persistently insufficient volume.

Data sampling was performed at 2.5 min intervals for NIBP and every 6 s for other physiological parameters and infusion control logs. Because vasopressor and fluid escalation triggers included the 2.5 min NIBP cycle, the maximum control response delay due to blood pressure assessment was approximately 2.5 min. In contrast, esSVI was continuously calculated, and its condition was evaluated every 6 s. No additional numerical filtering or smoothing was applied beyond the device’s internal quality control algorithms (e.g., BIS signal quality index [SQI] and esCCO). Automatic adjustment was discontinued and switched to manual administration when any of the following criteria were met: five consecutive hypotensive readings (mean arterial pressure < 65 mmHg), continuous infusion at 1200 mL/h for 30 min, total infusion volume exceeding 10 mL/kg, or infusion exceeding 15 mL/kg in the preceding hour. Once switched to manual control, automatic adjustment was not resumed.

This trial was single-blind (subject-blinded). Due to the need for intervention and safety monitoring, datasets were maintained with group label information attached. Therefore, blinding of the randomization assignment was not implemented for the statistical analysts. However, data quality assurance was performed through monitoring, auditing, and direct inspection procedures. Furthermore, the primary endpoints and statistical analysis plan were specified in advance in the study protocol, and no changes were made to them.

### 2.3. Efficacy Endpoints

The primary endpoint was the proportion of time, during the period from start to end of anesthesia (the evaluation period), that Pm was maintained at ≥65 mmHg. Secondary endpoints included the proportion of time that esSVI was below the threshold during the evaluation period, total fluid volume and rate per hour administered during the evaluation period, total phenylephrine dose and number of administrations during the evaluation period, urine output and blood loss, Ea value, and the average estimated stroke volume variation (esSVV) value during the evaluation period.

### 2.4. Safety Endpoints

(1) All adverse events observed in patients from the start of intraoperative observation to the end of postoperative observation were recorded, regardless of their causal relationship with the investigational device.

(2) Adverse events observed in persons other than patients (such as anesthesiologists or other medical staff) from device delivery to its retrieval were recorded only if causally related to the investigational device.

(3) Any malfunctions of the investigational device identified from its delivery to its retrieval were recorded.

### 2.5. Sample Size

Regarding anesthesia management using automatic control of propofol, remifentanil, and rocuronium, there is prior clinical trial [4] evidence validating the non-inferiority of the automatic group compared with the manual group. The mean value (standard deviation) for the primary endpoint in the automatic group was 87.21%. In addition, according to a previous study [5], the median proportion of time when mean arterial pressure was at least 65 mmHg (25th–75th percentile) was 100.0% (100.0–100.0%) in the computer-controlled group and 98.1% (95.0–98.8%) in the manually controlled group, with a difference (automatic group–manual group) of 1.9 percentage points.

Therefore, based on a comprehensive review of the results from the above prior clinical trials and previous studies, we assumed that the mean difference in the primary outcome for this study, namely, the proportion of time during the evaluation period in which the average blood pressure was maintained at 65 mmHg or higher (automatic group minus manual group), would be approximately 5%. Given a mean difference of 5% between the groups, a standard deviation of 15% for each group, a non-inferiority margin of 5%, a one-sided significance level of 2.5% (α error 2.5%), and a power of 80% (β error 20%), the required sample size to evaluate non-inferiority in this study was calculated using R (version 4.2.2), resulting in 36 participants per group [9]. Assuming a dropout rate of 10%, the target sample size was set at 40 participants per group, for a total of 80 participants.

### 2.6. Statistical Analysis

The comparison of the proportion of time during the evaluation period, from the start to the end of anesthesia, which was the primary evaluation item, when Pm was maintained at ≥65 mmHg, was examined for non-inferiority of automatic group compared with manual group. The difference in means (automatic group − manual group) was calculated, and the lower limit of the one-sided 97.5% confidence interval was derived using Student’s t-distribution assuming equal variances. Non-inferiority was concluded if the lower limit exceeded the non-inferiority margin of −5%. For esSVI, a secondary evaluation item, non-inferiority of automatic group compared with manual group was examined. The difference in means (automatic group − manual group) was calculated, and the upper limit of the one-sided 97.5% confidence interval was derived using Student’s t-distribution assuming equal variances. Non-inferiority was concluded if the upper limit fell below the non-inferiority margin of 5%. For other secondary evaluation items, Student’s *t*-test was used to test between the two groups. The incidence rate of adverse events (safety evaluation) was calculated using Fisher’s exact probability *p*-value (two-sided).

## 3. Results

**Figure 2:**
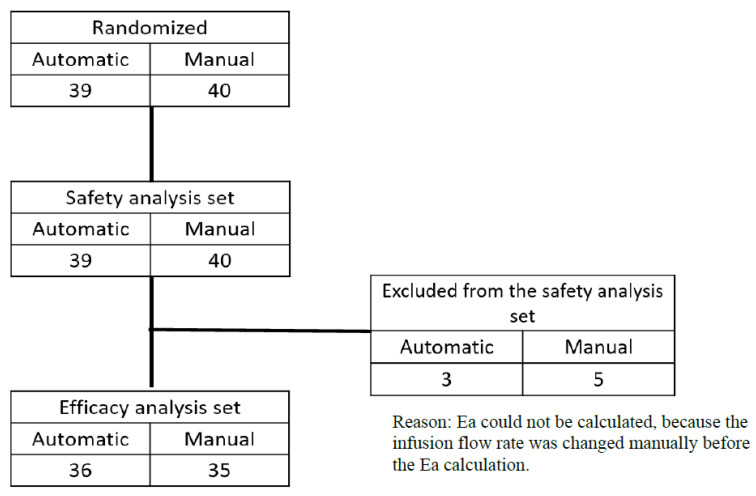
Flowchart of patients in study.

A total of 79 cases (40 in the manual group and 39 in the automatic group) were included in the safety analysis population. Of these, 8 cases (5 in the manual group and 3 in the automatic group) did not have efficacy evaluation data after randomization and were therefore excluded from the efficacy analysis population. As a result, 71 cases (35 in the manual group and 36 in the automatic group) were included in the efficacy analysis (Figure 2).

Patients’ background characteristics are shown in Table 1. There were no significant differences between the two groups in age, sex, height, weight, ASA-PS, or surgery duration.

The results of the efficacy assessment are shown in Table 2. The proportion of time during the evaluation period that mean blood pressure was maintained at 65 mmHg or higher, the primary endpoint, was 79.9 ± 15.7% in the M group and 82.0 ± 12.7% in the automatic group, demonstrating non-inferiority of the automated regulation group compared with the manual group (difference [automatic group − manual group]: 2.0 percentage points; one-sided 97.5% CI lower limit: −4.7%; non-inferiority margin: −5%). Regarding the secondary endpoint, the proportion of time during the evaluation period when esSVI was below the threshold was 55.8 ± 42.2% in the manual group and 33.0 ± 37.3% in the automatic group, demonstrating non-inferiority of the automatic group relative to the manual group (difference [automatic group − manual group]: −22.9 percentage points; one-sided 97.5% CI upper limit: −4.0%; non-inferiority margin: 5%). The total volume of infusion administered during the evaluation period and the amount administered per hour and per body weight were 1054 ± 659 mL and 4.9 ± 1.7 mL/kg/h in the manual group, and 949 ± 494 mL and 5.0 ± 1.8 mL/kg/h in the automatic group, respectively, with no significant differences between the two groups (*p* = 0.45 and *p* = 0.71, respectively). The total dose of phenylephrine used during the evaluation period was 2.1 ± 1.9 mg in the manual group and 1.7 ± 1.1 mg in the automatic group, with no significant difference between the groups (*p* = 0.23). The number of single doses of phenylephrine administered was 17 ± 17 times in the manual group and 16 ± 11 times in the automatic group, again with no significant difference (*p* = 0.65). Urine output was 226 ± 155 mL in the manual group and 210 ± 221 mL in the automatic group, with no significant difference (*p* = 0.73). Blood loss was 60 ± 106 mL in the manual group and 79 ± 213 mL in the automatic group, with no significant difference (*p* = 0.63). The Ea value was 2.0 ± 0.7 mmHg/mL in the manual group and 1.9 ± 0.5 mmHg/mL in the automatic group, with no significant difference between the groups (*p* = 0.27). The mean esSVV value during the evaluation period was 14.2 ± 6.8% in the manual group and 12.4 ± 5.1% in the automatic group, with no significant difference (*p* = 0.20). The mean esCCI value during the evaluation period was 1.9 ± 0.6 L/min/m^2^ in the manual group and 2.1 ± 0.5 L/min/m^2^ in the automatic group, with a significantly higher value in the automatic group (*p* = 0.04). The mean esSVI value (mean ± standard deviation) during the evaluation period was 34.4 ± 9.4 mL/m^2^ in the manual group and 39.0 ± 8.9 mL/m^2^ in the automatic group, also significantly higher in the automatic group (*p* = 0.04). An esSVV value of less than 13% suggests a high probability of adequate preload, but there was no significant difference between the manual and automatic groups: 49.6 ± 43.4 and 66.3 ± 36.2, respectively (*p* = 0.07). Similarly, the proportion of time with esSVI less than 30 mL/m^2^ was 27.6 ± 40.3% in the manual group and 19.2 ± 35.2% in the automatic group, with no significant difference (*p* = 0.35). However, the proportion of time with esCCI less than 2 L/min/m^2^ was 60.5 ± 41.2% in the manual group and 40.8 ± 41.2% in the automatic group, significantly longer in the manual group (*p* = 0.05).

The results of the safety evaluation are shown in Table 3. Adverse events during intraoperative and postoperative observation periods occurred in 31 cases (77.5%) in the manual group and 36 cases (92.3%) in the automatic group, with no significant difference between the groups (*p* = 0.11). Notably, no adverse events were observed intraoperatively; all occurred during the postoperative observation period. Of these, postoperative pain was the most common, with no severe adverse events or events causally related to the investigational device.

During the study period, a switch was made from automatic to manual control in 5 of the 39 patients in the automatic group. This was done because Pm was less than 65 mmHg and blood pressure did not increase sufficiently even with five consecutive infusions of phenylephrine according to the algorithm. These switches were based on the safety criteria set in advance; with priority on ensuring patient safety, automatic control was discontinued, and a switch was made to manual control by the anesthesiologist. In all cases in which a switch was made, no adverse events or device malfunctions were seen, and there were no problems in terms of patient safety.

There were three cases of intraoperative device malfunction, all involving rocuronium not being administered automatically as intended, though none was due to a defect in the investigational device itself. In the first patient, an occlusion alert occurred in the rocuronium syringe pump, causing automatic cessation of delivery because the pump motor reversed rotation and cleared the cumulative dose; as the surgery was near completion, no further administration was required. In the second patient, muscle relaxation could not be maintained because propofol backflow prevented rocuronium from reaching the patient despite syringe movement; administration was stopped as the surgery was ending. In the third patient, body movement occurred because the TOF electrode was incorrectly applied, preventing accurate TOFC measurement; rocuronium administration was switched from automatic to manual. None of these events affected patient safety.

The protocol stipulated that switching from automatic to manual control prohibited the subsequent reuse of automatic control. Furthermore, in the cases analyzed, no instances of crossover or protocol deviation occurred after switching from automatic to manual control.

## 4. Discussion

This study demonstrated that a computer-assisted hemodynamic management system was non-inferior to standard hemodynamic management administered by an anesthesiologist in low-risk surgery among patients with predicted blood loss of 1000 mL or less and an ASA performance status of 1–2. This system automatically administers vasoconstrictors and intravenous fluids when blood pressure drops, enabling rapid recovery. This is expected to prevent various complications caused by hypotension. Consequently, it reduces the burden on anesthesiologists and enhances productivity. It also helps prevent human errors such as administering the wrong medication or incorrect dosage, thereby improving patient safety.

In this study, to standardize anesthesia methods between the manual group and the automatic group, a total intravenous anesthesia (TIVA) system (AsisTIVA: Nihon Kohden) that automatically controls three drugs (analgesics, sedatives, and muscle relaxants) was used. This eliminated subjective influences such as the anesthesiologist’s preference and avoided effects due to differences in anesthetic dosage. Furthermore, in both groups, when mean blood pressure fell below 65 mmHg, phenylephrine was automatically administered to standardize the effect of restoring afterload on blood pressure.

Since CO is the product of SV and HR, and Ea can be approximated as the product of HR and TPR (Equations (1) and (2)), blood pressure was calculated as the product of SV and Ea (Equation (3)), thereby enabling SV to reflect changes in blood volume.

Pm = CO·TPR(3)

Ea = HR·TPR(4)

Pm = SV·Ea(5)

**Figure 3:**
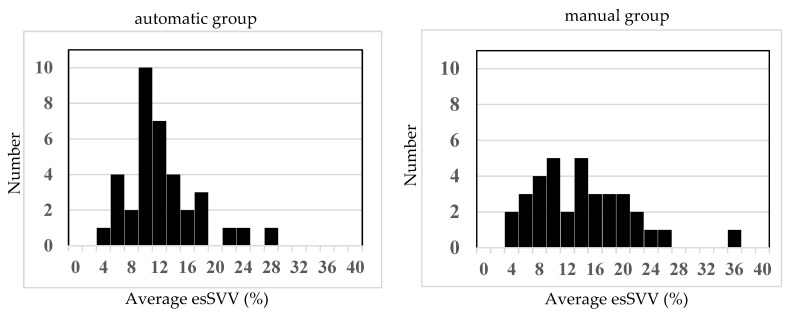
Histograms of mean esSVVvalues. Histograms showing the distribution of estimated stroke volume variation (esSVV) during anesthesia. The manual group shows a wide distribution (4–26%), suggesting greater variability in volume status. In contrast, the automatic group shows a narrower distribution centered around 10–12%, indicating more stable maintenance of esSVV within the optimal range, which may reflect better volume control.

The lower normal limit for preload in the present study was determined as follows. First, the initial value of effective arterial elastance (Ea) was calculated from the Pm and SV before induction of anesthesia. Next, using this state, the SV when Pm was 65 mmHg was determined (Equation (3)), and that value (esSVI65) was used as a threshold. As a result, the proportion of time with Pm maintained at 65 mmHg or higher was 79.9 ± 15.7% in the manual group and 82.0 ± 12.7% in the automatic group, demonstrating non-inferiority of the automatic group to the manual group in maintaining Pm ≥ 65 mmHg (difference [the automatic group − the manual group]: 2.1 percentage points; one-sided 97.5% CI lower limit: −4.7%; non-inferiority margin: −5%). The absolute difference in the primary endpoint (82.0% in the automated group vs. 79.9% in the manual group) was only 2.1 percentage points, which may not be clinically significant. However, both groups maintained a mean arterial pressure ≥ 65 mmHg for the majority of the anesthetic period, in accordance with current recommendations for intraoperative blood pressure management. Statistical analysis demonstrated non-inferiority of the automated group compared with the manual group, suggesting that automated fluid management provides clinical efficacy comparable to that of manual management. The total administered phenylephrine was 2.1 ± 1.9 mg and 1.7 ± 1.1 mg, respectively, with no significant difference, and the infusion rate per hour was 4.9 ± 1.7 mL/kg/h and 5.0 ± 1.8 mL/kg/h, also with no significant difference. Thus, although phenylephrine was automatically administered for hypotension, fluid adjustment in response was shown to be non-inferior in the automatic group, which was managed automatically, compared with the manual group, in which anesthesiologists administered fluids per the ERAS protocol. Taken together, this suggests that, when hypotension results from decreased afterload and preload is maintained, since SV tends to increase, there is no need to increase fluids, and this was appropriately assessed. In addition, even without bleeding, if capacitance vessels dilate, causing relative hypovolemia and hypotension, SV decreases, requiring increased fluid administration, which was also appropriately assessed. Furthermore, when comparing the time during which esSVV was maintained below 13%, it was 49.6 ± 43.3% in the manual group and 66.7 ± 36.2% in the automatic group, with large variability, and there was no significant difference. As shown in the histogram in Figure 3, values in the manual group were widely distributed from 4% to 26%, but those in the automatic group were maintained in a relatively narrow range centered around 10–12%, suggesting that blood volume was kept within the optimal range in the automatic group. However, with an increase in afterload leading to elevated blood pressure, SV decreases, and if it falls below the threshold, there is the possibility of fluids being increased despite high blood pressure.

The computer-assisted hemodynamic management system developed by Joosten et al. [5,6] sets individualized MAP targets during surgery and provides computer assistance to maintain this blood pressure. In the system we developed, the lower limit of MAP is set at 65 mmHg from NIBP measurements, and the system is activated when MAP below 65 mmHg is measured. Then, with a closed loop system, the system monitors Ea and SVI over time as afterload and preload, respectively. We did not use CO as an indicator in this study. The reason is that CO changes not only in accordance with changes in preload, but is also affected by changes in afterload. Similarly, neither SVV nor PPV is affected by preload alone. However, Ea is an indicator of afterload that is unaffected by preload, so it was selected as the control variable with the thought that it may improve the control accuracy. In our system, the values set for Ea and SVI when the patients enter the operating room are used. When measured values are above the set values, control is done simply by administering infusion at a maintenance dose. Thus, for cases when invasive arterial pressure measurements are unnecessary, and there is little blood loss, low surgical invasiveness, and no problems with cardiac function, this system is intended to raise and maintain blood pressure when it decreases by quickly maintaining afterload and administering infusions as needed.

The completely automated hemodynamic management system adopted by Joosten et al. requires invasive arterial pressure measurements, so the system is difficult to activate during the induction of anesthesia when hypotensive states are frequent. Moreover, since multiple factors affect MAP, an ideal computer-assisted hemodynamic management system should obtain information not only on the current blood pressure, but also on intravascular volume, depth of anesthesia, indicators of sympathetic nervous system inhibition, heart rate, and cardiac function. Thus, while the closed loop system of Joosten et al. can rigorously control MAP, it cannot be relied on alone to ensure appropriate management of hemodynamics. There are other reports of closed-loop resuscitation of hemorrhagic shock with fluid, but since more time is needed for the introduction of this to clinical settings, these are only reports of the experimental stage [10,11]. The advantage of our fluid management infusion system is not that it is a completely automated hemodynamic management system, but that it is a system specifically for monitoring hypotension; as such, it can be introduced into usual clinical settings. It also constantly monitors hypotension and responds immediately during the induction of anesthesia, or when multiple anesthesia tasks are being performed, so a shorter duration of hypotension and a reduction in human errors can be expected. In addition, since invasive arterial pressure measurements are not required, it has the major advantage of being able to operate non-invasively from before the induction of anesthesia.

The algorithm used in this study is considered useful for hypotension, but during hypertension, there is a potential risk of fluid overload, as an upper limit for blood pressure was not set in this study. In this study, no malfunctions or operational errors were observed with the newly developed equipment. Though no safety concerns were identified, the small number of cases requires further evaluation of additional cases. Regarding the automatic control of vasopressors, the same research team has previously demonstrated the non-inferiority of the automatic group compared with the manual group [12]. In the future, we plan to combine the current automatic fluid management system with this automatic control of vasopressors in further studies.

In this study, we used esCCO, the accuracy of which may be inferior to that of gold-standard techniques (thermodilution, transesophageal echocardiography). In past studies, however, exCCO was reported to have a high accuracy similar to intermittent bolus thermodilution cardiac output [13,14], and it is thought that the non-invasive measurement method of esCCO can also be used clinically. In addition, signal quality is constantly monitored using the signal quality index (SQI) of esSVI. An alert is given when quality declines, when there is an abnormal measurement value, or when there is a device malfunction. Patient safety is ensured by switching to manual control as needed. Data accuracy is guaranteed by comparison with the raw data and monitoring/inspection. Further verification of the accuracy of esSVI and the limitations of the system in special cases will be needed.

### Limitations

This study has the following limitations. First, in this study, cardiac contractility was assumed to be normal and constant. If cardiac contractility increases and blood pressure rises, stroke volume (SV) will increase, so it is expected that the infusion rate will decrease. However, in the case of hypotension due to heart failure, SV decreases, and the infusion volume increases; therefore, care must be taken to avoid fluid overload. Second, in accordance with the ERAS guidelines, preoperative dehydration was prevented, and cases with a predicted blood loss of less than 20 mL/kg were included in the study. Therefore, this device is not applicable for cases with concomitant dehydration symptoms or surgeries involving hemorrhagic shock. In such cases, rapid and massive fluid or blood transfusion is required, but the maximum flow rate of the current infusion pumps is 1200 mL/h, which may be insufficient to meet the required volume. Third, in this system, the infusion rate is increased only after the mean arterial pressure (Pm) drops below 65 mmHg, and the system does not incorporate a function to proactively increase the infusion rate in anticipation of a drop in blood pressure. By incorporating a preventive infusion function for cases where a decrease in blood pressure is anticipated, it is expected that the accuracy of blood pressure management could be improved. In addition, this study did not include a detailed analysis of the variance in fluid administration patterns between groups. Therefore, whether the automated system responds more consistently to similar hemodynamic challenges compared with conventional management remains unclear.

Fourth, this study was limited to cases classified as ASA 1,2, non-hemorrhagic surgery, and without blood transfusion. Therefore, the performance of this system in cases with unstable hemodynamics or hemorrhagic conditions remains unknown. Future large-scale, multicenter, validation studies including complex surgeries and patients with cardiac dysfunction are considered necessary.

Fifth, we did not investigate pneumoperitoneum and positioning effects or the surgical category (examples: laparoscopic surgery and open surgery). We also did not analyze hemodynamic stability during critical surgical moments (incision, retraction, position changes) in this study. These effects will need to be investigated in the future.

Sixth, this study had a small sample size and was single-blinded, so there is a possibility that bias occurred. Large-scale research is needed in the future.

In conclusion, evaluation of automatic infusion volume control software for automatic fluid administration demonstrated non-inferiority for the primary endpoint of efficacy, and there were no safety issues with the use of the test device.

## Figures and Tables

**Table 1:** Characteristics of study participants.

Variable	Automatic (*n* = 39)	Manual (*n* = 40)	*p* Value
Age (y)	57.1 ± 18.5	62.4 ± 12.3	0.14
Male sex, *n* (%)	12 (30.8%)	13(32.5%)	1.00
Weight (kg)	58.9 ± 12.2	58.1 ± 13.8	0.76
Height (cm)	158.2 ± 7.0	159.0 ± 8.8	0.63
Body mass index (kg/m^2^)	23.5 ± 4.3	22.8 ± 4.5	0.49
No. of patients ASA-PS I/II	11/28	11/29	1.00

ASA-PS: American Society of Anaesthesiologists Physical Status.

**Table 2:** Comparisons of the primary and secondary outcomes.

Variable	Automatic (*n* = 36)	Manual (*n* = 35)	Difference (CI Limit) *p* Value
Duration of MBP > 65 mmHg (%)	82.0 ± 12.7	80.0 ± 15.7	2.0 (−4.7) ^1^
Duration of SVI < esSVI65 (%)	33.0 ± 37.3	55.8 ± 42.2	−22.9 (−4.0) ^2^
Fluid infusion (mL/kg/h)	5.0 ± 1.8	4.9 ± 1.7	0.71 ^3^
Total dose of phenylephrine (mg)	1.7 ± 1.1	2.1 ± 1.9	0.23 ^3^
Number of single doses of phenylephrine (time)	16± 11	17± 17	0.65 ^3^
Total blood loss (mL)	79 ± 213	60 ± 106	0.63 ^3^
Total urine output (mL)	210 ± 221	226 ± 155	0.73 ^3^
Ea value (mmHg/mL)	1.9 ± 0.5	2.0 ± 0.7	0.27 ^3^
Average of esSVV (%)	12.4 ± 5.1	14.2 ± 6.8	0.20 ^3^
Average of esCCI (L/min/m^2^)	2.1 ± 0.5	1.9 ± 0.6	0.04 ^3^
Average of esSVI (mL/m^2^)	39.0 ± 8.9	34.4 ± 9.4	0.04 ^3^

^1^ Difference is Automatic–Manual. CI limit is one-sided 97.5% CI lower limit. Non-inferiority is concluded if the lower limit exceeds the non-inferiority margin of −5%. ^2^ Difference is Automatic–Manual. CI limit is one-sided 97.5% CI upper limit. Non-inferiority is concluded if the upper limit falls below the non-inferiority margin of 5%. ^3^ *p* value: Student’s *t*-test (two-tailed). MBP: mean blood pressure. Ea: effective arterial elastance. esSVI: estimated stroke volume index. esSVV: estimated stroke volume variation. esCCI: estimated continuous cardiac index.

**Table 3:** Safety evaluation.

	Automatic (*n* = 39)	Manual (*n* = 40)	*p* Value
All adverse events that occurred intraoperatively, n (%)	0	0	
All adverse events that occurred within 48 h postoperatively, n (%)	36 (92.3%)	31 (77.5%)	0.114
Pain	32 (82.1%)	30 (75.0%)	
Nausea	5 (12.8%)	3 (7.5%)	
Vomiting	4 (10.3%)	5 (12.5%)	
Chills	3 (7.7%)	2 (5.0%)	
Shivering	1 (2.6%)	0	
Others	4 (10.3%)	0	
Adverse events that occurred intraoperatively and had a causal relationship with the investigational device, n (%)	2(5.1%)	1 (2.5%)	
Adverse events that occurred within 48 h postoperatively and had a causal relationship with the investigational device, n (%)	0	0	

*p* value: Fisher’s exact test (two-tailed).

## Data Availability

The datasets used and/or analyzed during the current study are available from the corresponding author upon reasonable request.
